# Complete mitochondrial genome and phylogenetic analysis of *Penicillium citrinum* in dark tea

**DOI:** 10.1080/23802359.2019.1637794

**Published:** 2019-07-12

**Authors:** Zhiyuan Hu, Liu Shiquan, Zhenggang Xu, Meng Dong, Suchun Liu

**Affiliations:** a College of Food Science and Technology, Hunan Agricultural University, Changsha, China;; b Hunan Provincial Key Lab of Dark Tea and Jin-hua, School of Materials and Chemical Engineering, Hunan City University, Yiyang, China

**Keywords:** *Penicillium citrinum*, mitochondrial genome, next generation sequencing, phylogenetic analysis

## Abstract

*Penicillium citrinum* is a common polluting microorganism in dark tea production. Our study was performed to report the complete mitochondrial genome of *P. citrinum*. The mitochondrial genome of *P. citrinum* was a circular DNA molecule of 27,537 bp in length, encoding 42 genes as follows: 15 PCGs, two rRNAs, 24 tRNAs, and an independent ORF. A (36.14%), T (37.06%), C (11.83%), and G (14.98%) was composed of genomic bases. In addition, phylogenetic analysis showed that *Penicillium* sp. exhibited a closest relationship with the taxonomic status of *P. citrinum*.

*Penicillium citrinum* is a filamentous fungus within the genus *Penicillium*. Its widely distributed and can grow in fruits, grains (Houbraken et al. 2010) and tea (Dutta et al. 2010; Haas et al. 2013); it can produce mycotoxin citrinin under certain conditions (Li et al. 2017), which has adverse effects on human kidney and immune system (Aiko and Mehta 2013; Föllmann et al. 2014). The present study reported the complete mitochondrial genome sequence of a *P. citrinum* isolated from dark tea, providing important information for population genetics, evolution and taxonomy of this fungus.

The sample (*P. citrinum*) was isolated from dark tea produced in Yiyang City, Hunan Province, China (N28°32′, E112°23′) and preserved in Hunan City University (JH1205). Extraction of mitochondrial genome from *P. citrinum* was realized by DNeasy Plant Mini Kit (Qiagen, Valencia, CA). Mitochondrial DNA sequencing was performed based on Illumina miseq 2500 platform (Illumina, San Diego, CA). Adapters and low-quality reads were removed using NGS QC Toolkit (Patel and Jain 2012). Gene annotation was further carried out using MITOS Web Server (Bernt et al. 2013), and the results were submitted to the GeBank database with accession number: MK919205.

The mitochondrial genome sequence of *P. citrinum* was 27,537 bp in length and it had 42 genes, including 15 protein-coding genes (PCGs), two ribosomal RNA genes (rrnLand rrnS), 24 transporting RNA (tRNA) genes, and an independent open reading frame (ORF349). Typical ATG was used as the initiator codon and typical TAA (rps3, cox1, atp9, cox2, nad4L, nad5, nad2, nad1, nad4, atp8, atp6, and nad6) and TAG (nad3, cob, and cox3) as the terminator codon in all the 15 protein-coding genes. The contents of four bases in the genome were A (36.14%), T (37.06%), C (11.83%), and G (14.98%), respectively, and the content of A + T was 74.2%, displaying a significant bias at AT position.

Furthermore, the phylogenetic tree (Figure 1) was constructed by cluster analysis of 17 species of related fungi using maximum-likelihood and neighbor-joining. In view of the phylogenetic tree, *Penicillium* sp. (Mardanov et al. 2016) was found to be the most closely related to the taxonomic status of the mitochondrial genome of *P. citrinum*. Collectively, the sequencing results of the mitochondrial genome of *P. citrinum* can be used for the further study of comparative genomics and the further study of systematic taxonomy of *Penicillium*.

**Figure 1.**
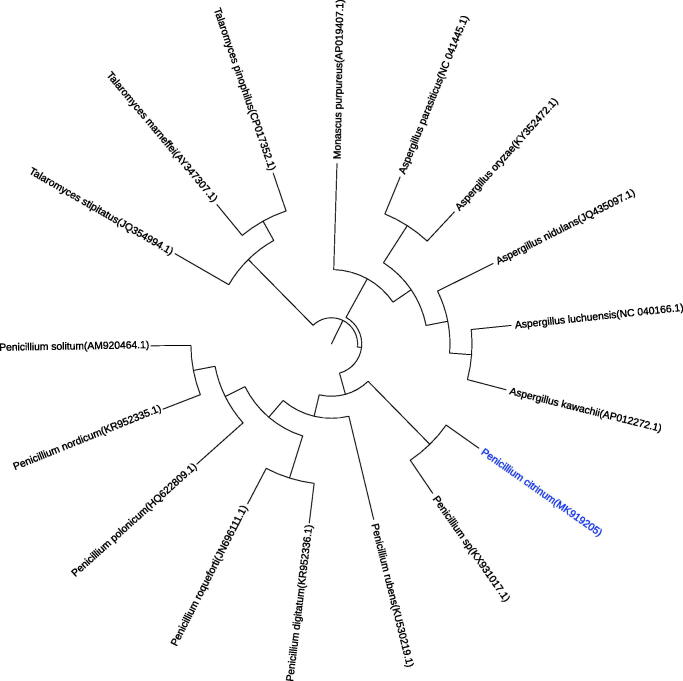
The phylogenetic tree based on eight *Penicillium* mitochondrial genome sequences, five *Aspergillus* mitochondrial genome sequences, two *Talaromyces* mitochondrial genome sequences, and one *Monascus* mitochondrial genome sequences. The neighbour-joining (NJ) phylogenetic tree was constructed with MEGA 7 (with 1000 bootstrap replicates).
